# Controlled Release of Pyrimidine Compound Using Polymeric Coated ZIF-8 Metal-Organic Framework as Glucagon-Like Peptide-1 Receptor Agonist Carrier

**DOI:** 10.3390/molecules25184313

**Published:** 2020-09-20

**Authors:** Shaikha S. AlNeyadi, Naheed Amir, Mohammad A. Ghattas, Noor Atatreh, Shaikha S. Alketbi, Ruba Al Ajeil, Abdu Adem

**Affiliations:** 1 Department of Chemistry, College of Science, UAE University, Al-Ain P.O. Box 15551, UAE; 201504873@uaeu.ac.ae (S.S.A.); 201540208@uaeu.ac.ae (R.A.A.); 2 Department of Pharmacology, College of Health and Science, UAE University, Al-Ain P.O. Box 17666, UAE; naheed_amir@uaeu.ac.ae (N.A.); abdu.adem@uaeu.ac.ae (A.A.); 3 College of Pharmacy, Al Ain University, Abu Dhabi P.O. Box 112612, UAE; mohammad.ghattas@aau.ac.ae (M.A.G.); chancellor@aau.ac.ae (N.A.); 4 Department of Pharmacology, College of Medicine and Health Sciences, Khalifa University, Abu Dhabi P.O. Box 127788, UAE

**Keywords:** GLP-1 agonist, antidiabetic, ZIF-8, pyrimidine, alginate

## Abstract

This work demonstrates synthetic strategies for the incorporation of a synthesized pyrimidine glucagon-like peptide-1 (GLP-1) agonist into alginate-coated ZIF-8. The prepared pyrimidine GLP-1 agonist used for the treatment of diabetes type II, was trapped inside polymer coated ZIF-8. The encapsulation of the GLP-1 agonist was confirmed by UV-visible and FT-IR spectroscopies. Furthermore, the release kinetics of GLP-1 agonist drug from alginate-coated ZIF-8 were investigated in phosphate-buffered saline at 37 °C at pH 8 and 1.5. The alginate-coated ZIF-8 exhibited much faster drug release at basic pH than at pH 1.5, indicating the potential of the alginate-coated ZIF-8 system to overcome the fast degradation at acidic pH of the stomach and improve the drug’s activity. This study may open the way for the synthesis of new metal organic frameworks (MOFs) to enhance drug delivery systems.

## 1. Introduction

Type 2 diabetes (DM2), a state of hormonal disruption and incretin deficiency, is gradually becoming a global epidemic [1]. Recent drugs used in the type 2 diabetes therapy have familiar limitations: They raise body weight and increase loss of β-cell function [2]. However, incretin-based treatments, which concentrate on GLP-1, have recently emerged and attracted much attention.

GLP-1 seems to be a goal for type-2 diabetes therapy due to its unique mechanism of action. GLP-1 is an incretin hormone formed by intestinal enteroendocrine L cells subsequent to nutrient digestion. Once the glucose levels are raised, GLP-1 endorses insulin secretion in the pancreas, and it has minimum effect when glucose levels are normal [3]. This glucose-dependent insulinotropic effect is mostly favorable for diabetes thereby because it reduces the hazards of hypoglycemia, which is one of the side effect of some anti-diabetes drugs [3]. GLP-1 also reduces glucagon secretion, additional helping in lowering glucose levels [4]. Some previous studies also have proposed that GLP-1 can enhance β-cell function and prevent β-cell apoptosis. Therefore, reduce the development of β-cell failure [4]. In the GI tract, GLP-1 slows gastric emptying, leading to reduce the postprandial glucose levels [5]. Also, by activating GLP-1 receptors in the nervous system, GLP-1 could improve satiety and prevent energy intake, which may help reduce the bodyweight [6].

In spite of its effective anti-diabetes properties, the clinical application of natural GLP-1 is hindered by its fast degradation by the dipeptidyl peptidase-4 enzyme in vivo, resulting in a half-life around 2 min [7]. The short half-life of GLP-1 has limited the function of native GLP-1 in the treatment of DM2. The effort to detect GLP-1 analogs has led to the development of the drugs exenatide [7] and liraglutide [8]. However, their route of administration as injections limits their clinical use. Therefore, orally active drug agonists of the GLP-1 receptor are urgently needed [9].

Diabetic patients require life-long treatment, and repeated drug administration reduces patient compliance and augments the frequency of side effects (e.g., diarrhea, abdominal discomfort, painful urination, and cramping) [5,10]. New formulation approaches for antidiabetic drugs are being tracked and can improve bioavailability, reduce dose administration frequency, and improve patient compliance [11]. Therefore, the development of a novel and efficient delivery system for therapeutic antidiabetic drugs is a prerequisite.

Many drug delivery systems have been well-known in the last two decades. Unlike conventional drug carriers such as micelles, liposomes, dendrimers, and mesoporous silica nanoparticles, MOFs are interesting delivery systems because of their ability to incorporate significant loadings of many active compounds either within their pores or within the hybrid network itself. Also, the release of the active compound can be achieved under physiological conditions through framework degradation, drug diffusion, and host–guest interactions [12].

Some of the most popular MOF therapeutic agents are zeolitic imidazolate framework (ZIF) series. ZIF-8 has excellent chemical stability in water and alkaline solution but rapid decomposes in acidic solution [13], which is a pH-sensitive release property. pH-responsive ZIF-8 has been an attractive candidate for controllable drug release [14]. In addition, zinc has potential as a novel therapeutic agent in diabetes. Many studies have shown that zinc metal has helpful effects in diabetes treatments. Additionally, zinc plays an important role in β-cell function, insulin action, glucose homeostasis, and the pathogenesis of diabetes and its complications.

ZIF-8 is identified for its fast degradation at acidic pH [15]. Therefore, it is essential to enhance its biostability for targeted delivery of drugs at higher pH. To overcome the limitations of ZIF-8 in biological applications and improve drugs’ activities, we decided to synthesize a polymer-coated ZIF-8. Alginic acid is a pH-sensitive natural polymer used in oral drug carriers. Its structure contains carboxylic acid and hydroxyl groups; therefore, at higher pH it expands owing to the loss of acidic protons and repulsion between negative oxygen ions [16]. Alginate-coated ZIF-8 is formed through an interaction between the carboxylate groups of alginate polymer and functional groups present in the linker.

**Scheme 1:**
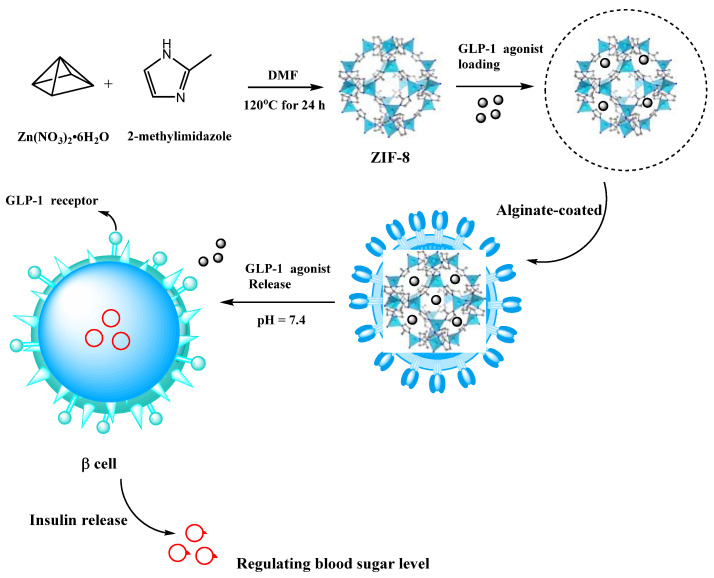
Schematic of the synthesis of alginate-coated zeolitic imidazolate framework (ZIF)-8 as a glucagon-like peptide-1 (GLP-1) agonist carrier and its antidiabetic activities.

The emergence of new drug delivery systems for antidiabetic drugs is a worldwide problem in clinical medicine. Considering the important need for modern targeted drug delivery and the several advantages of ZIF-8 as a carrier in controlled-release applications, such as its highly specific surface area and porous structure, we describe the synthesis of a polymer-coated ZIF-8 as a carrier for our synthesized pyrimidine GLP-1 agonist. This system may exhibit reduced cytotoxicity, improved absorbance, and sustained release of the GLP-1 agonist, which would avoid frequent monitoring of glucose levels and multiple doses and may result in synergistic effects with the Zn metal ion in the ZIF-8 formulation (Scheme 1). The sample was examined by powder X-ray diffraction (XRD), thermogravimetric analysis (TGA), scanning electron microscopy (SEM), and Fourier transform infrared (FT-IR) analyses. We believe that this study elucidates the use of MOFs as drug delivery systems and specifically their potential supportive roles in antidiabetic treatments.

## 2. Results and Discussion

### 2.1. Chemistry

**Scheme 2:**
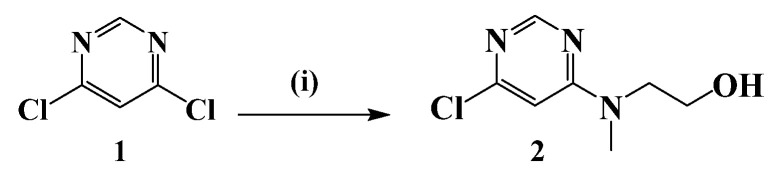
Reagents and conditions for the synthesis of compound **2**: 4,6-dichloropyrimidine (**1**, 1.0 mol); 2-(methylamino)]ethanol (1.0 mol); *N,N*-diisopropylethylamine (DIPEA) (1.1 mol), ethanol, 120 °C, 150 W.

We described a highly regioselective amination of 4,6-dichloropyrimidine which strongly favors the formation of the C4-isomer using microwave-assisted synthetic protocol for the preparation of 4-substituted pyrimidines in excellent yields at reaction times significantly shorter (~10 min) than previously reported method [17]. The synthesized compounds were characterized and evaluated for their in vitro and in vivo antidiabetic activities. Among all tested compounds, compound **2** showed promising antidiabetic activity. Compound **2** was synthesized as shown in Scheme 2. Firstly, 4,6-dichloropyrimidine (**1**) was transformed into the corresponding 4-substituted pyrimidine **2** using a microwave protocol. Compound **1** was reacted with amine in the presence of *N,N*-diisopropylethylamine (DIPEA) as a base in a simple nucleophilic reaction in ethanol to afford the desired pyrimidine derivative **2.** The product was isolated in a crystalline form in 93% yield. The nucleophilic substitution of the chlorine atom at C-4 of the pyrimidine ring was facilitated using DIPEA, which enhanced the nucleophulicity of the amine via the removal of one proton from the amino group. The amine anion attacks the chorine atom to form the targeted 4-substituted pyrimidine derivative **2**.

The structure of the prepared compound **2** were established using spectroscopic methods. The IR spectrum of compound **2** showed signals at ν = 3100 and 2898 cm^−1^, which were attributed to stretching vibrations of the aromatic and aliphatic C-H groups. The -OH stretching vibration appeared at 3390 cm^−1^. The ^1^H-NMR spectrum of compound **2** showed signals at δ = 2.87 ppm, which were assigned to hydroxy groups, while the signals at δ = 3.10 ppm were attributed to methyl protons. The signals at δ = 3.77 and 3.84 ppm were assigned to two CH_2_ groups. Protons H_5_ and H_2_ of the pyrimidine ring appeared as singlets, one at δ = 6.46 ppm and one at δ = 8.32 ppm, respectively. On the other hand, the ^13^C-NMR spectrum of compound **2** showed three new signals at δ = 31.1, 52.7 and 61.0 ppm, which were assigned to the aliphatic carbons. Signals at δ = 162.9, 159.5, 157.4 and 101.6 ppm were attributed to C_4_, C_6_, C_2_, and C_5_ on the pyrimidine ring, respectively.

### 2.2. Antidiabetic Activity

#### 2.2.1. Pharmacological Evaluation of the Compound **2** on Fasting Blood Glucose (FBG)

**Figure 1:**
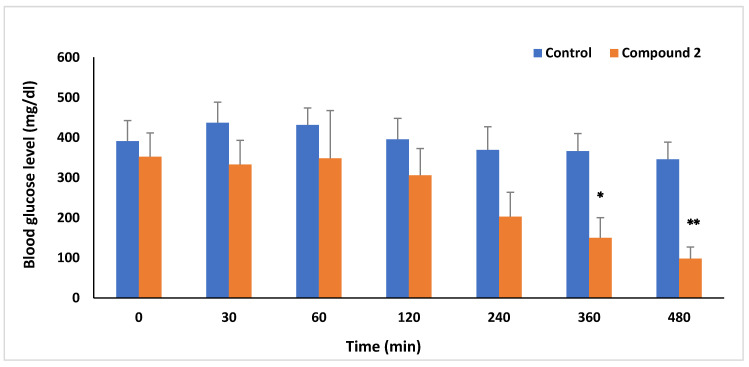
Antihyperglycemic effect of test compound **2** on the fasting blood glucose level of streptozotocin (STZ) diabetic rats. The results are shown as the means ± SEMs; *n* = 8 rats; * *P* < 0.05, ** *P* < 0.01 vs. the STZ diabetic control group.

In this study, rats injected with (STZ) showed significant increases in plasma glucose levels and kidney weight along with decreases in serum insulin and body weight in comparison with these values in nondiabetic rats (results not shown). These symptoms indicated the development of diabetes was characterized by chronic and persistently elevated plasma glucose levels. STZ induces diabetes by selectively destroying insulin-producing pancreatic endocrine cells. Decreased body weight in STZ-induced diabetic rats is believed to be caused by dehydration and breakdown and catabolism of fats and proteins. Increased catabolic activity upon the administration of STZ resulted in muscle wasting and subsequent body weight loss. Compound **2** was orally administered to the treated groups of diabetic rats at a dose of 1.0 µM/kg. The glucose levels in their blood were followed over 8.0 h. Compound **2** resulted in significant reductions in blood glucose levels at 6 and 8 h after treatment compared to the levels in diabetic control animals (Figure 1).

#### 2.2.2. Effects of the Compound **2** on Insulin Secretion in the βTC6 Cell Line

**Figure 2:**
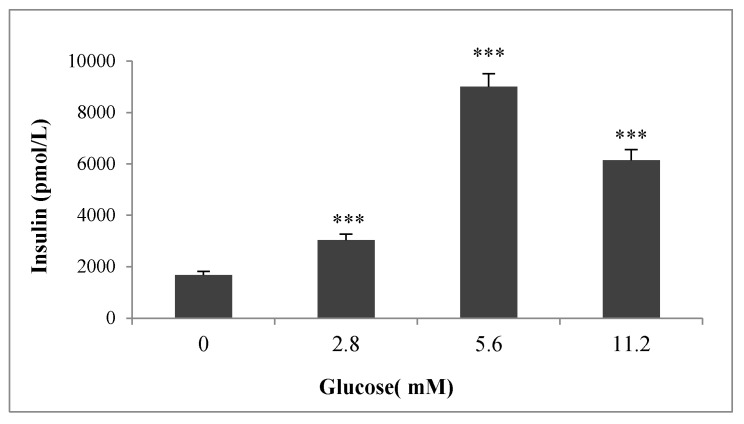
Glucose response graph of βTC6 cells without administration of the drug, *** *P* < 0.001.

Using the high-range insulin sandwich ELISA kit defined in the experimental part, the secretion of insulin by βTC6 cells was evaluated. Figure 2 shows the glucose response curve of βTC6 cells in the absence of tested compound. Glucose (2.8 mM) administration gave a mild insulin response of approximately 3000 pmol/L and was used in testing both exenatide (reference drug) and the compound **2** that showed antidiabetic *in vivo* effects in rats.

**Figure 3:**
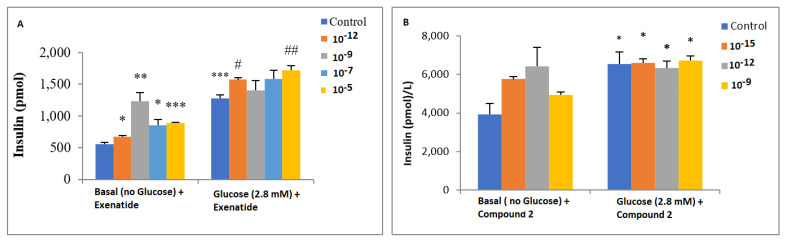
(**A**)The effects of exenatide (10^−12^–10^−5^ M) and (**B**) compound **2** (10^−15^–10^−^^9^ M) on insulin secretion in βTC6 cells in the absence (basal) and presence of 2.8 mM glucose. Results are means of triplicates ± SEMs; * *P* < 0.05, ** *P* < 0.01, *** *P* < 0.001 from relative basal control and # *P* < 0.05, ## *P* < 0.01, ### *P* < 0.001 from glucose 2.8 Mm.

Treatment of the βTC6 cell line with standard drug exenatide caused in a significant increase in insulin secretion compared to basal secretion. Furthermore, in the presence of 2.8 mM glucose, the exenatide at the concentration 10^−15^ and 10^−12^ M significantly improved insulin secretion compared to that of the control (2.88 mM glucose alone) (Figure 3A). Figure 3B shows the effects of the potent compound **2**, at concentration 10^−15^, 10^−12^, and 10^−9^ M on insulin secretion in the absence and presence of 2.8 mM glucose. In the absence of glucose and at concentration 10^−15^, 10^−12^, and 10^−9^ M, compound **2** significantly increased insulin secretion compared to that of the basal control. In the presence of 2.8 mM glucose, the insulin secretion for the different concentrations of **2** displayed no significant alteration from that of the control (2.8 mM glucose alone). Compound **2,** in which the chlorine atom at position 4 is replaced by 2-(methylamino)ethanol, provided a significant increase in the degree of the response, telling that the hydrogen bond donor is favored at this position and that the length of the linker affects binding to the ago-allosteric binding site of the GLP-1 receptor.

**Figure 4:**
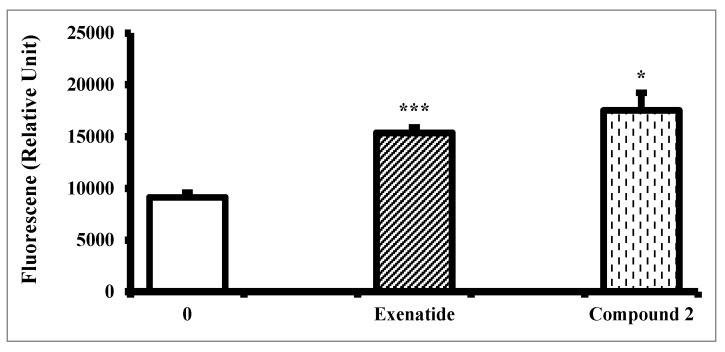
Effects of test compound **2** (10 µM) on glucose uptake by βTC6 cells. The results are the means of triplicates ± SEMs; * *P* < 0.05, *** *P* < 0.001 vs. control (zero concentration); * *P* < 0.05, *** *P* < 0.001 vs. standard drug (exenatide).

In addition, compound **2** treatment resulted in the glucose uptake shown in the Figure 4.

### 2.3. Docking of Compound **2** in the GLP-1 Receptor

The whole GLP-1 receptor has never been fully crystallized; instead, only the extracellular domain structure has been solved by Runge et al [18]. In addition, described antidiabetic pyrimidine compound are supposed to exert their agonistic effect on the GLP-1 receptor via an allosteric mechanism [19]. Such agonistic activity was shown even on a truncated version of the GLP-1 receptor that lacks the extracellular domain (Δ-ECD-GLP-1) [19]. This is why docking of pyrimidine compound **2** should be conducted on the Δ-ECD-GLP-1 receptor and not on Runge’s solved extracellular structure.

**Figure 5:**
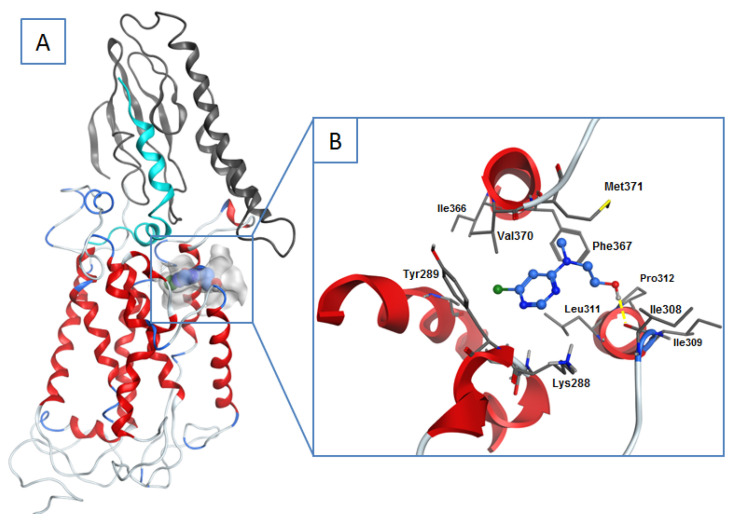
(**A**) 3D structure of the full GLP-1 receptor is shown along with GLP-1 (cyan ribbon), the docked pose of compound **2** (blue balls and sticks) and the suggested allosteric pocket (gray surface). The extracellular domain that was truncated during docking is shown as a dark gray ribbon. (**B**) The binding mode of compound **2** (blue balls and sticks) docked into the suggested binding site of the Δ-ECD-GLP-1 receptor (gray sticks). The picture was generated by MOE [21]. Hydrogen bonding is shown by the yellow dotted line.

The complete 3D structure of the GLP-1 receptor and binding modes of some GLP-1 agonists were predicted and validated by Lin and Wang [20] by homology modeling, molecular docking and long-term molecular dynamics simulation on a lipid bilayer. This model has been validated by the newly revealed crystal structure of the extracellular domain of GLP-1 and was suggested to serve as a reference for characterizing interactions between GLP-1 and its agonists. Cpd1 was assumed to be a noncompetitive agonist and was docked onto the GLP-1 receptor by Lin and Wang [20]. In their molecular dynamics simulations, cpd1 was found to be fairly stable in an allosteric pocket at the top surface of the transmembrane domain, near the GLP-1 binding site. Sloop et al [19] showed that two pyrimidine/quinoxaline-based compounds act on the GLP-1 receptor as allosteric agonists. The compound **2** activated the truncated GLP-1 receptor and was believed to act within or near the transmembrane domain of the GLP-1 receptor, similar to cpd1 [19]. Since our pyrimidine-based agonist **2** is thought to exert its agonistic activity in the same way as cpd1, it is justifiable that we dock our compound into the same allosteric pocket of cpd1 using the truncated part (Δ-ECD-GLP-1) of the homology-modeled receptor provided by Lin and Wang [20] (Figure 5).

The binding energies of compound **2** docking into the cpd1 allosteric pocket of the Glp-1 receptor showed that compound **2** exhibited a favorable binding mode, as shown by it having the lowest docking energy score (Glide XP score = −5.1 kcal/mol) and highest ligand efficiency score (>−0.43 kcal/mol). The ligand efficiency score considers the docking score in relation to the ligand size and thus suggests that this compound can be good lead that might eventually be developed into therapeutically useful antidiabetic agent. This result is consistent with our pharmacological result, where compound **2** showed antidiabetic property.

Figure 5 shows the 3D structure of the full GLP-1 receptor along with GLP-1 (cyan ribbon), the docked pose of compound **2** (blue balls and sticks) and the suggested allosteric pocket (gray surface). The extracellular domain that was truncated during docking is shown as a dark gray ribbon. Figure 5b shows the binding mode of compound **2** (blue balls and sticks) docked into the suggested binding site of the Δ-ECD-GLP-1 receptor (gray sticks). The pyrimidine ring of compound **2** appears buried in a hydrophobic pocket, forming van der Waals interactions with the side chains of Lys288, Leu311, Tyr289, and Val370. Additionally, the primary hydroxyl group in compound **2** forms a hydrogen bond with the backbone amide of the Ile308 residue (shown as yellow dotted lines), providing further stabilization of the ligand–receptor complex.

Thus, our findings are consistent with the results of molecular dynamics simulations performed by Lin and Wang on cpd1, where the Tyr289 and Val370 residues seemed to play a vital role in stabilizing cpd1 within the allosteric pocket [20]. Allosteric binding of this type of agonist was suggested by molecular dynamics simulations to hinder the movement of the transmembrane domain, shifting the GLP-1 receptor equilibrium from the inactive form to the active form [20]. Although these predictions should be confirmed by experimental biochemical work, they can still help in understanding and designing potent GLP-1 receptor agonists.

We report a low-molecular-weight pyrimidine compound **2** that activates the GLP-1 receptor to induce glucose-dependent insulin secretion both in vitro and in vivo. This molecule may offer a therapeutic advantage because the compound can act alone or in combination with GLP-1.

To enhance the bioavailability of compound **2,** the synthesized GLP-1 agonist (compound **2**) was encapsulated in prepared alginate-coated ZIF-8 to optimize the drug loading efficiency, allow gradual drug release, protect the drug from degradation in physiological media before it reaches the targeted site and avoid its toxic effects on the body.

### 2.4. ZIF-8 Characterization

#### 2.4.1. Synthesis

**Scheme 3:**
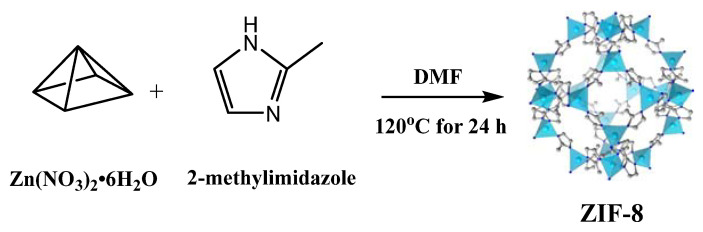
Synthesis of ZIF-8.

In this work, ZIF-8 was prepared by a DMF solvothermal method using zinc nitrate hexahydrate and 2-methylimidazole [15] (Scheme 3). It was definite before that in the ZIF-8 structure, zinc atoms were connected through nitrogen atoms by 2-methylimidazolate linkers to form MOFs, generating nanosized pores formed by four-, six-, eight-, and twelve-membered rings of tetrahedral ZnN_4_ clusters. For the production of porous MOF-based materials, it is essential to conduct solvent exchange process to help in purification of the ZIF-8 frameworks. The synthesized ZIF-8 sample was therefore immersed in additional MeOH at room temperature for 3 days. In this step, DMF molecules were replaced by more weakly-interacting MeOH molecules that would be simply removed under vacuum in the following activation step.

Samples were characterized by powder XRD, TGA, SEM and FT-IR spectroscopy as follows.

#### 2.4.2. XRD Analysis

**Figure 6:**
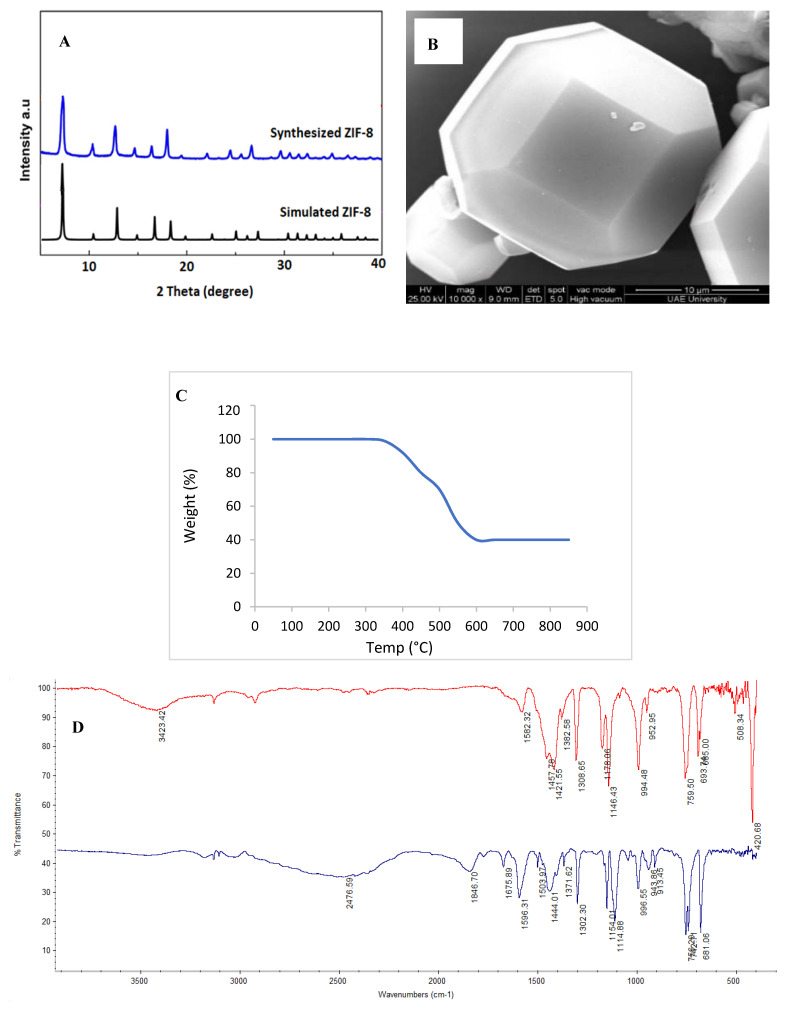
Characteristics of ZIF-8: (**A**) XRD pattern, (**B**) SEM image, (**C**) TGA diagram, and (**D**) IR spectrum.

Figure 6a demonstrates the XRD pattern of ZIF-8 nanoparticles. A sharp peak below 10° (with a 2θ of 7.13°) was detected in the XRD diffractogram of ZIF-8, indicating that a highly crystalline material was obtained (Figure 6a). The peak position and the characteristic diffraction peaks of ZIF-8 structure were 7.13° (011), 10.7° (002), 12.5° (112), 14.3° (022), 16.1° (013), 17.8° (222), 24.1° (233), and 26.2° (134). Both position and intensity of the diffraction peaks are in line with those defined previously in the literature [22,23], which confirms that ZIF-8 was synthesized effectively.

#### 2.4.3. SEM Analysis

Figure 6b displays the morphology of the synthesized sample. The pure ZIF-8 sample had rhombic dodecahedron morphologies with an average particle size of 1 mm, which matched the results in the literature [24], indicating that the synthesis occurred correctly. An SEM result of a synthesized ZIF-8 nanoparticle is shown in Figure 6b. The dried ZIF-8 nanoparticles were monodispersed. Apparently, the configuration of ZIF-8 nanoparticles can be altered if the molar ratio of metal ion to ligand is altered [25]. For example, at molar ratio (1:2), rhombic dodecahedron crystals will be formed, while ZIFs can change into truncated rhombic dodecahedrons when the molar ratio of metal ion to ligand is moderately low such as 1:4 or 1:8. The molar ratio in our study is 1:2, so the expected configuration of the ZIF-8 will be rhombic dodecahedrons. Furthermore, the anions coordinated with the metal ions can directly affect the types of chemical bonds in ZIFs. It can be probable that the formed ZIFs are of medium size (<1.0 μm) if the metal ions are nitrates. Subsequently the metal salt used in our study was zinc nitrate, the sizes of the ZIF-8 structure ranged from 0.1 to 1.0 µm.

#### 2.4.4. Thermogravimetric Analysis (TGA)

Figure 6c displays a TGA curve for ZIF-8 nanoparticles. A mild weight loss occurred from 100 to 300 °C. This weight loss may be caused by the escape of guest molecules such as 2-methylimidazole and gas molecules from the cavity. The main weight loss of ZIF-8 takes place at 350–500 °C and can be due to the decomposition of the 2-methyl imidazole ligand. In this work, it was found that ZIF-8 could afford Langmuir surface areas of up to 1700 m^2^/g, as reported previously in the literature. Certainly, many ZIF-8 samples with surface areas ranging from 1300 m^2^/g to 1810 m^2^/g were previously produced using solvothermal methods [15].

#### 2.4.5. FT-IR Spectroscopy Analysis

The structure of ZIF-8 was studied by using FT-IR analysis as shown in Figure 6d. ZIF-8 showed bands at 3423, 3135, 2929, 1582, 1458, 1421, 1382, 1308, 1146, 994, 759, 693, and 420 cm^−1^. These FT-IR peaks were in agreement with those earlier described [26]. The FT-IR spectrum of ZIF-8 was significantly different from that of 2-methylimidazole (Figure 6d). In the FT-IR spectrum of 2-methylimidazole, a band ranging from 3400 cm^−1^ to 2200 cm^−1^ with a maximum at around 2476 cm^−1^ was detected, the presence of a N-H…N hydrogen bond. Additionally, an interaction between the N-H….N bending (out of plane) and N-H stretching as by a peak at 1846 cm^−1^ (Figure 6d) [16]. These important bands (2476 cm^−1^ and 1846 cm^−1^) of 2-methylimidazole disappeared in ZIF-8 spectrum, representing that the 2-methylimidazole linkers were deprotonated in the ZIF structure. Moreover, absorption peaks at 3135 cm^−1^ and 2929 cm^−1^ due to the stretching vibrations of C-H bonds in the methyl group and the imidazole ring were detected in the spectra of ZIF-8. In addition, these bands overlapped with the absorption peaks of the N-H stretching vibration in the spectrum of 2-methylimidazole. The bands at 994 and 759 cm^−1^ were allocated to a C-N bending vibration and a C-H bending mode, respectively. The peak at 693 cm^−1^ was attributed to the ring out-of-plane bending vibration of the 2-methylimidazole ring. Remarkably, the Zn-N stretching vibration band was observed at 420 cm^−1^, suggesting that zinc ions chemically connect the nitrogen atoms of the methylimidazole groups to form imidazolate [27].

**Figure 7:**
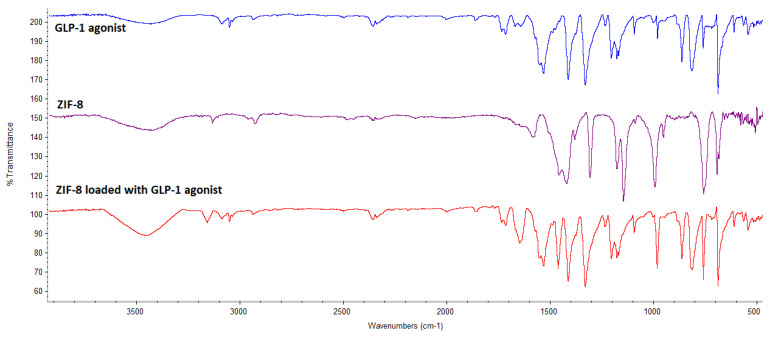
IR spectra for GLP-1 agonist, ZIF-8 and ZIF-8 loaded with GLP-1 agonist.

**Figure 8:**
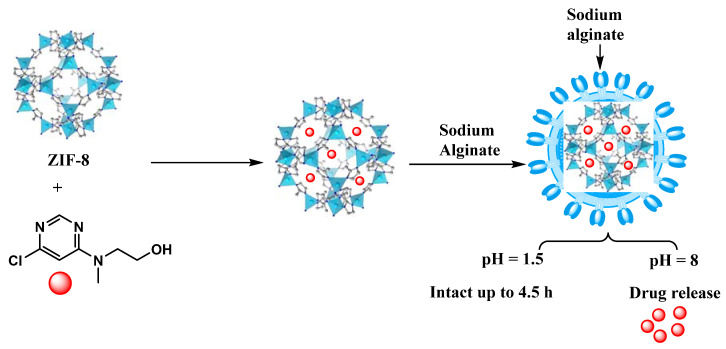
Drug loading-release process.

To produce the drug-loaded ZIF-8, the ZIF-8 nanoparticles were immersed into a drug solution for 24 h. After centrifugation, the solution was studied by UV-Vis spectroscopy. The results displayed that the drug loading efficiency was 85%. In addition, the loading was measured by FT-IR analysis. As shown in Figure 7, the peaks in the FT-IR spectra of drug-loaded ZIF-8 established the positive encapsulation of the drug inside the ZIF-8 structure. The short stability of ZIF-8 nanoparticles and fast decomposition under acidic medium limit their use in biological media. Therefore, the prepared ZIF-8 framework was coated with alginate polymer to enhance its stability in acidic conditions. This will save the drug loading into the ZIF-8 structure to pass from the stomach acidic environment, and release in the basic intestine medium. The drug-loaded ZIF-8 nanoparticles were immersed in an aqueous solution of alginate for 1 h to coat them (Figure 8).

**Figure 9:**
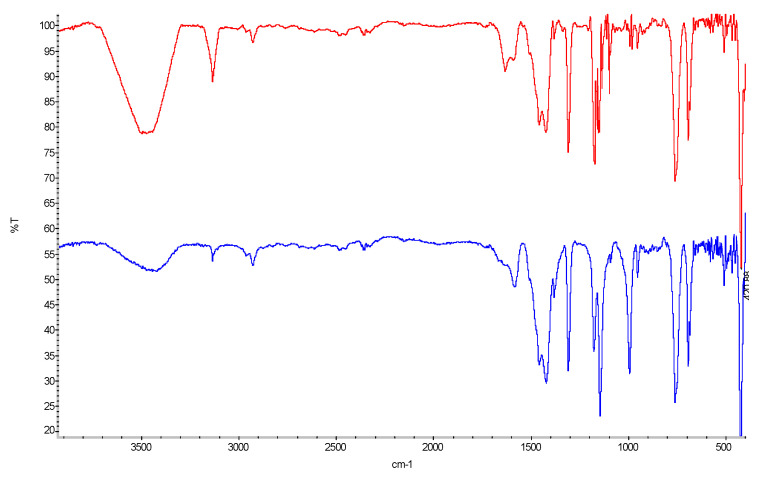
FT-IR spectra of ZIF-8 (bottom) and alginate-coated ZIF-8 (top).

The prepared ZIF-8 and alginate-coated ZIF-8 was established by FT-IR analysis (Figure 9). The FT-IR spectrum of ZIF-8 structure show absorption peaks at 3135 cm^−1^ and 2929 cm^−1^ due to the aromatic and aliphatic C-H stretching vibrations of the imidazole moiety, respectively. The peaks at 1582 cm^−1^ are associated to the stretching vibration of C=N groups. The stretching mode of the Zn-N bond shows at 420 cm^−1^. The peaks between 1100 and 1400 cm^−1^ results from C-N stretching vibrations. The FT-IR spectrum of coated ZIF-8 (Figure 9) show that in addition to the absorption bands of ZIF-8, a broad band resonates at 3474 cm^−1^, which due to the O-H vibrations of alginate polymer. The peaks at 3134 cm^−1^ due to stretching vibrations of aliphatic C-H groups of alginate and ZIF-8. The peak at around 1633 cm^−1^ correspond to stretching vibrations of carboxylate groups. The stretching peak of C-O shows at 1097 cm^−1^. It was supposed that the alginate and the ZIF-8 are attached by hydrogen bonding and the attraction between the zinc cation and the negative carboxylate groups of the alginate molecules.

**Figure 10:**
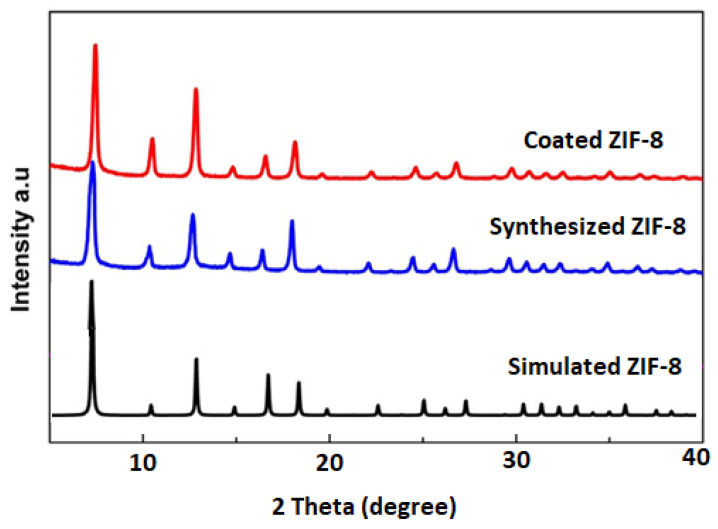
XRD patterns of simulated ZIF-8 (black), synthesized ZIF-8 (blue) and alginate-coated ZIF-8 (red).

According to Figure 10, the coating procedure didn’t affect the crystallinity of ZIF-8 structure, which remained intact.

**Figure 11:**
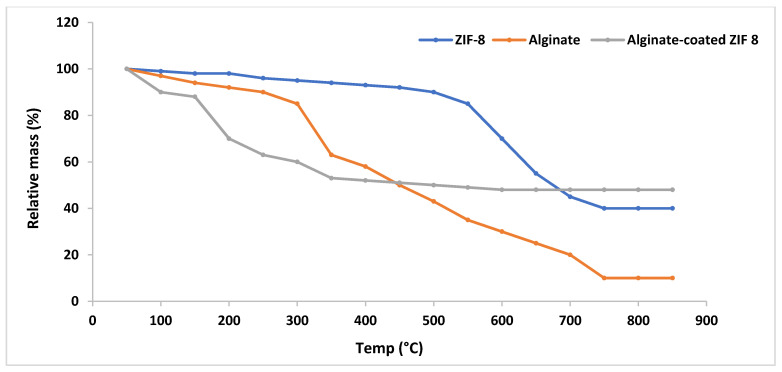
TGA curve of ZIF-8 (top), alginate (middle), and alginate-coated ZIF-8 (bottom).

For additional evaluation of the prepared alginate-coated ZIF-8 structure, TGA was measured as shown in Figure 11. The thermal features of ZIF-8 was consistent with those described in the literature [28]. According to the results, the weight loss of the ZIF-8 structure began at around 350 °C; however, a rapid weight loss happened when the temperature raised from 450 °C to 800 °C, which is associated to the decomposition of the organic framework components. The coated ZIF-8 framework showed weight loss starting at 250 °C due to the decomposition of the alginate molecules. Further weight loss (25 wt.%) was completed at around 350 °C.

**Figure 12:**
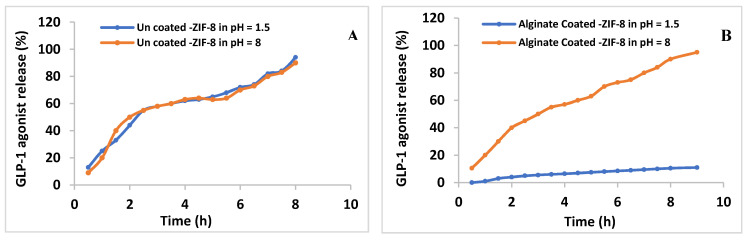
Effect of the uncoating (**A**) and coating (**B**) ZIF-8 nanoparticles on drug release from ZIF-8 structure. Compound **2** was released from coated ZIF-8 framework in PBS at a virtual gastric pH (1.5, bottom) and a virtual intestine pH (8, top) at 37 °C.

Prior to the drug release study, a calibration curve was determined for compound **2** in PBS after dissolving it in 1 mL of 20 mM NaOH. The calibration curve was generated from the absorption at 340 nm, where the drug showed maximum absorption. To examine the effect of polymeric coating on the stability and the release performance of GLP-1 agonist-loaded ZIF-8 nanoparticles at two pH values, the ZIF-8 nanoparticles were immersed in pH 1.5 PBS solution and in pH 8 PBS solution for around 24 h. The release curves obtained by UV-Vis spectroscopy (Figure 12), shows nearly 90% of the drug load was released from the coated ZIF-8 in a precise way for 9 h in PBS at pH 8. Remarkably, only 8.5% of the loaded compound **2** was released during the initial first 6 h at pH 1.5. Therefore, the alginate coating protected the ZIF-8 structure very efficiently from degradation in acidic medium. As predicted, ZIF-8 nanoparticles will pass through acidic gastric environment without significant degradation and release their drug load in the small intestine.

It is consequently predictable that the coated ZIF-8-loaded drug can securely pass through the stomach and the drug is released in the small intestine. The polymeric coating will help in controlling drug release as follow: In an acidic environment, the carboxylic groups in alginic acid are neutral, and therefore no repulsive force between these molecules, while in basic media, the carboxylic groups will lose their acidic proton and converted to carboxylate ions. Consequently, the alginate coating expands because of the negatively charged carboxylate ions repelling each other and the trapped drug molecules can then be released. This will help to reduce the harmful side effects of the drug in the stomach. Coated drug-loaded ZIF-8 has an excellent ability to store compound **2** and control its release in basic intestinal media.

## 3. Material and Methods

### 3.1. General

All reagents and chemicals were purchased from Sigma-Aldrich (St. Louis, MO, USA) and used without further purification. Thin-layer chromatography (TLC) was performed on silica gel glass plates (Silica gel, 60 F_254_, Fluka, Merck, Darmstadt, Germany). Column chromatography was performed on Kieselgel S (silica gel S, 0.063–0.1 mm, Merck, Darmstadt, Germany). Melting points were recorded on a Gallenkamp apparatus (Toledo, OH, USA), and were uncorrected. Infrared spectra were calculated using KBr pellets on a Thermo Nicolet model 470 FT-IR spectrophotometer (Thermo Scientific, Waltham, MA, USA). Nuclear magnetic resonance spectra (NMR) were recorded using a Varian-400 MHz spectrometer (^1^H-NMR at 400 MHz and ^13^C-NMR at 100 MHz; Agilent Technologies, Santa Clara, CA, USA) using CDCl_3_ as solvent. Tetramethylsilane (TMS) was used as internal reference and chemical shifts are stated as part per million; (δ values, ppm). Elemental analysis was performed on a Leco Model CHN-600 elemental analyzer (Ontario, Canada). Microwave synthesis was performed using the CEM microwave system (Matthews, USA). Absorption measurements were carried out using an Agilent 8453 spectrophotometer (Santa Clara, USA) supported with 1.0 cm quartz cells (Varian, Austria). XRD analysis was performed using a Shimadzu-6100 powder XRD diffractometer (Shizmadu-series, Kyoto, Japan) using Cu-Kα radiation with λ = 1.542 Å. Diffraction data were collected within the 2θ range of 20–80° at a rate of 1°/min. For TGA, a 0.2 g sample was heated to 600 °C at a rate of 5°/min. The weight was monitored as a function of temperature. SEM micrographs were taken using a FEI SEM Quanta Inspect S50 scanning electron microscope (Bruker Nano GmbH, Germany) operated at an accelerating voltage of 15–30 kV and equipped with an EDS-Oxford INCA PENTA system for determining the elemental distribution on the surface of the sample. Surface area and pore size study was performed using a PMI’s BET Sorptometer (BET-201-AEL, PMI, USA).

### 3.2. Synthesis of the GLP-1 Agonist

#### 2-[(6′-Chloropyrimidin-4′-yl)(methylamino)]ethanol (**2**)

Compound **2** was prepared by mixing 4,6-dichloropyrimidine (1.0 mmol, 0.148 g) and 2-(methylamino)ethanol (1.0 mmol) in ethanol (3 mL) at 0 °C in the presence of *N,N*-diisopropyl ethylamine (DIPEA, 1.1 mmol) under microwave irradiation for 10 min. The progress of the reaction was checked by TLC. Then, ethyl acetate (10.0 mL) was added to the reaction mixture, and the pH was adjusted to 7 using 6 M HCl. The mixture was washed with a saturated aqueous solution of NaHCO_3_. The organic layer was dried over anhydrous MgSO_4_. The excess solvent was removed under reduced pressure, and the produced residue was purified by column chromatography using ethyl acetate-hexane (1:1) to afford the final product **2** in 93% yield; white solid, mp 138 °C; IR (KBr, cm^−1^): 3390 (O-H), 3100 (C-H, aromatic), 2898 (aliphatic C-H), 1670 (C=N), 1414 (C-N); ^1^H-NMR (400 MHz, CDCl_3_) δ ppm: 2.87 (s, OH, exchanges with D_2_O), 3.10 (s, 3H, CH_3_), 3.77 (m, 2H, CH_2_), 3.84 (m, 2H, CH_2_), 6.46 (1H, H_5_-pyrimidine), 8.32 (s, 1H, H_2_-pyrimidine); ^13^C-NMR (100 MHz, CDCl_3_) δ ppm: 162.9 (C4-pyrimidine), 159.5 (C6-pyrimidine), 157.4 (C2-pyrimidine), 101.6 (C5-pyrimidine), 61.0 (CH_2_), 52.7 (CH_2_), 37.1 (CH_3_); anal. calcd for C_7_H_10_ClN_3_O: C, 44.81; H, 5.37; N, 22.40; found: C, 44.93; H, 5.44; N, 22.71.

### 3.3. Synthesis of MOFs

#### 3.3.1. Synthesis of ZIF-8

ZIF-8 was prepared solvothermally using DMF, and a substrate mixture was prepared using 0.34 g (1 mmol) of zinc nitrate hexahydrate (Zn(NO_3_)_2_·6H_2_O) and 0.167 g (2 mmol) of 2-methylimidazole in 50 mL of *N,N*-dimethylformamide (DMF), which was stirred until a clear solution was obtained. This solution was introduced to a 100 mL Duran bottle with a Teflon-taped screw cap and then heated at 140 °C for 24 h in a convection oven. The product was filtered, washed with DMF several times, kept in MeOH for 3 days and then dried at room temperature.

#### 3.3.2. Synthesis of GLP-1 Agonist-Loaded ZIF-8

ZIF-8 Nanoparticles (50 mg) were charged in 10 mL of a 400 mg/L GLP-1 agonist (compound **2**) aqueous solution. The solution was stirring at 22 °C for 24 h and then the solution centrifuged for approximately 10 min. The drug concentration was calculated using UV-Vis spectroscopy analysis and the GLP-1 agonist load was calculated using the below equation:

(1) $$\mathrm{GLP}\text{-}1\;\mathrm{agonist}\;\mathrm{wt}.\%=\frac{\mathrm{GLP}\text{-}1\;\mathrm{agonist}\;\left(\mathrm{mg}\right)}{\mathrm{MOF}\;\left(\mathrm{mg}\right)}\%$$

where the amount of the drug trapped inside the MOF pores is 3.4 mg of 4 mg. In order to coat the drug-loaded ZIF-8 nanoparticles with alginate polymer, 13 mg of GLP-1 agonist-loaded ZIF-8 were immersed in 10 mL of 3 g/L alginate solution. The mixture was stirred at 22 °C for 1 h. Then, the solution was centrifuged at 10,000× *g* for 10 min, and analyzed by UV-Vis spectroscopy to identify the amount of the drug was leached from the ZIF-8 during the coating. Finally, in vitro release of the GLP-1 agonist from the coated ZIF-8 was achieved by placing the coated ZIF-8 nanoparticles in phosphate-buffered saline (PBS) at 37 °C under stirring. The concentration of the GLP-1 agonist released from the nanoparticles was calculated at different times. When a specific amount of the solution was taken for investigation, the same amount replaced by of fresh buffer. After steady intervals, samples were removed and centrifuged, and the solutions were collected for UV-Vis analysis at a wavelength of 340 nm. This procedure was carried out for the coated ZIF-8 nanoparticles. The GLP-1 agonist concentration in each sample was calculated using the calibration curve that was determined earlier. The corrected concentration of released GLP-1 agonist was calculated using the following equation [29]:

(2) $$Ctcorr=Ct+\frac{v\sum_{0}^{t-1}Ct}{V}$$

where *C_tcorr_* is the adjusted concentration at time *t*, *Ct* is the calculated concentration of GLP-1 agonist at time *t*, *v* is the volume of the derived samples, and *V* is the total volume of release solution.

#### 3.3.3. Synthesis of Polymer-Coated ZIF-8-Compound **2**

ZIF-compound **2** nanoparticles were added to a solution of sodium alginate at a 2:1 nanoparticle:alginate ratio. The solution was placed at 22 °C under stirring. After 3 h, the solution was centrifuged at 10,000× *g* for 10 min, and the coated nano ZIF-8 was washed with water twice to eliminate the free alginate particles that were not bound to the ZIF-8 surface. The nanoparticles were dried and analyzed by TGA to determine the amount of alginate that coated the nano ZIF-8, while the FT-IR and X-ray analysis were used to examine the effect of the coating on particle crystallinity.

### 3.4. Antidiabetic Activity

#### 3.4.1. Animal Housing and Diabetic Induction

Rats were housed under standard environmental conditions (22 ± 2 °C, 65 ± 5% humidity and 12-hr light/12-hr dark cycles) and sustained with free access to water and an ad libitum standard laboratory diet. Four rats were housed in each cage to provide sufficient space and avoid unnecessary morbidity and mortality. Diabetes was induced by intraperitoneal injection with freshly prepared streptozotocin (STZ) in citrate buffer, pH 4.5, at a dose of 60 mg/kg. Diabetes was verified after 48 h using an ACCU-CHEK Performa glucometer. Rats with stable fasting blood glucose levels over 350 mg/dL from 4–6 weeks after STZ injection were measured diabetic.

#### 3.4.2. In Vivo Testing

Diabetic rats were divided into a control (*n* = 8) and treated groups (*n* = 8). All animals were fasted overnight, and their fasting blood glucose (FBG) level was measured in blood from the tail vein by using an ACCU-CHEK Performa glucometer. The animals received either vehicle (0.0002% DMSO in distilled water) or test compound. The blood glucose levels were measured at 30, 60, 90, 120, 240, 360, and 480 min after the dose. The rats were allowed to drink water only during the experiment.

#### 3.4.3. In Vitro Testing

##### Cell Culture

Cells of βTC6, a mouse immortalized insulin-secreting pancreatic β cell line (T-SV40), were grown in DMEM culture medium containing 25.0 mM glucose, 1.0 mM sodium pyruvate, 4.0 mM L-glutamine, 44.0 mM sodium bicarbonate, 15.0% (*v*/*v*) FBS, and 50.0 μg/mL gentamicin in a 5.0% CO_2_ incubator at 37 °C. The medium was replaced every 48 h with fresh culture medium, and cells were subcultured as necessary to prevent overconfluence. Cells were passaged by treatment with 0.25% trypsin and 0.91 mM EDTA at passages 6–8.

##### Insulin Secretion Assay

βTC6 cells (1 × 10^5^ cells/mL) were cultured in a 24-well plate for 48 h in a 5% CO_2_ incubator at 37 °C. The cells were then preincubated for 30 min in modified Krebs/Ringer buffer (KRB) (118.5 mM NaCl, 25 mM NaHCO_3_, 4.74 mM KCl, 1.19 mM MgSO_4_, 2.54 mM CaCl_2_, 10 mM HEPES, 1.19 mM KH_2_PO_4_, 0.1% BSA, pH 7.4) in a CO_2_ incubator. The resultant cells were washed with fresh buffer and incubated for another 30 min. Solutions of the chloropyrimidine compound **2** (10^−9^–10^−15^ M) were prepared by diluting stock standard solutions with KRB. Solutions containing 0.000004% DMSO were obtained. Then, 250 μL of the solutions of different concentrations were added to the cells, which were then incubated in a 5% CO_2_ incubator at 37 °C for 120 min in the absence or presence of a 2.80 mM glucose solution. The total reaction volume was 1 mL for each experiment. To maintain a total volume of 1 mL, either 750 µL or 500 µL of KRB was added first, followed by 250 µL of 4X concentrated compound and glucose solutions (basal experiment: 750 µL of KRB + 250 µL of 4X test drug; glucose stimulation experiment: 500 µL of KRB + 250 µL of 4X 2.8 mM glucose + 250 µL of 4X test drug). After incubation, the supernatant layers were collected and subjected to sandwich ELISA using a high-range insulin assay kit according to the manufacturer’s instructions. As per instructions from the manufacturer of the kit, 10 µL samples were incubated with enzyme conjugate solutions on shaker plates for 2.0 h at room temperature. The plates were washed, 3,3′,5,5′-tetramethylbenzidine (TMB) was added, the plates were incubated for 15 min, and the reaction was stopped. The absorbance intensity of the solutions was read at 450 nm with a Tecan microplate reader. The sensitivity of the insulin ELISA was 216 pmol/L. The average intra- and interassay coefficients of variation were 3.37 and 2.29%, respectively. The levels of insulin were expressed as pmol/L.

##### Cell-Based Glucose Uptake Assay

Cayman′s Glucose Uptake Cell-based Assay Kit (Cayman Chemical, Ann Arbor, Michigan, USA) was used as a tool for studying modulators of cellular glucose uptake. The kit employs 2-[*N*-(7-nitrobenz-2-oxa-1,3-diaxol-4-yl)amino]-2-deoxyglucose (2-NBDG), a fluorescently labeled deoxyglucose analog, as a probe for the detection of glucose taken up by cultured cells. βTC6 cells were seeded onto a 96-well clear flat bottom black plate at 5 × 10^4^ cells/mL and allowed to adhere overnight at 37 °C and 5% CO_2_ in tissue culture medium (DMEM containing 25 mM glucose, 1 mM sodium pyruvate, 4 mM L-glutamine, 44.05 mM sodium bicarbonate (NaHCO_3_), 15% (*v*/*v*) FBS, and 50 μg/mL gentamicin). The complete medium was then removed, and the cells were washed with KRB (118.5 mM NaCl, 25 mM NaHCO_3_, 4.74 mM KCl, 1.19 mM MgSO_4_, 2.54 mM CaCl_2_, 10 mM HEPES, 1.19 mM KH_2_PO_4_, 0.1% BSA, pH 7.4). Conditioning of the cells proceeded at 37 °C and 5% CO_2_ twice for 30 min each. The conditioning buffer was then removed and replaced with 450 μM 2-NBDG with 10 µM either test compound or 10 µM reference drug in KRB. The cells were then incubated further at 37 °C and 5% CO_2_ for 10 min to allow them to endocytose the glucose analog. At the end of the treatment, the plate was centrifuged for 5 min at 400× *g* at room temperature. The 2-NBDG in basal medium was then removed, and the cells were washed with 200 µL of Cell-Based Assay Buffer. The plate was centrifuged for 5 min at 400× *g* at room temperature. Then, 100 µL of Cell-Based Assay Buffer was added to each well after aspiration of the supernatant. The amount of 2-NBDG taken up by cells was measured immediately using excitation and emission wavelengths of 485 nm and 535 nm, respectively, in a Tecan infinite M200 microplate reader.

##### Statistical Analysis

Experimental results were expressed as the mean ± SEM and statistically assessed by SPSS-20. Differences between test animal and control values were evaluated using Student’s t-test.

##### Ethical Approval

Approval for this project was obtained from the Faculty of Medicine and Health Sciences Ethics Committee, United Arab Emirates University. The study protocol was approved by the Animal Ethics Committee of the United Arab Emirates University with the approval ID No.: RECA: A10-12.

### 3.5. Docking Studies

The three-dimensional structure of the whole GLP-1 receptor was modeled with molecular modeling by the Lin and Wang [20] group. This model has been validated by the recently revealed crystal structure of the extracellular domain of the GLP-1 receptor. It was also suggested to serve as a valuable reference for characterizing the interactions between the GLP-1 receptor and its agonists. The receptor extracellular domain (ECD) of the GLP-1 receptor was truncated. The truncated structure, consisting of the intracellular and transmembrane parts of the GLP-1 receptor (Δ-ECD-GLP-1 receptor), was checked for any missing atoms using the protein preparation module in MOE [30]. Partial charges on each atom and the protonation state of each ionizable group of truncated structure were processed via the Protein Preparation Wizard [31] in Maestro software [32]. On the other hand, the LigPrep module [33] in the Maestro program [34] was used to prepare the ligands and generate the dominant ionization state for ionizable functional groups.

Using docking and molecular dynamics, Lin and Wang predicted an allosteric binding pocket for a thiadiazole-based GLP-1 receptor agonist, 2-[6,7-dichloro-3-(trifluoromethyl)naphthalene-2-ylthio]-5-methyl-1,3,4-thiadiazole [20]. This pocket was identified using the 3D coordinates of 2-(6′,7′-dichloro-3′-(trifluoromethyl) naphthalene-2′-yl)thio)-5-methyl-1,3,4-thiadiazole (cpd1), and a grid box was created using the Receptor Grid Generation module in Glide [35] The hydroxyl and thiol groups present in the active site were set as rotatable. Since compound 2 was confirmed to activate the Δ-ECD-GLP-1 receptor, our compound **2** was docked to the above allosteric binding site predicted by Lin and Wang for the cpd1 agonist. Docking was performed using Glide software [35], where the extra-precision (XP) algorithm [36] was employed for conformational sampling. Subsequently, docked poses were scored via the Glide XP scoring function, which includes terms for van der Waals interactions, hydrogen bonds, electrostatic interactions, desolvation penalties, and penalties for intraligand contacts [36].

## 4. Conclusions

The objective of the present investigation was to develop drug delivery formulation for antidiabetic targeted delivery of GLP-1 agonist. In the present study, the findings revealed that the drug-loaded ZIF-8 coated by alginate, a low-cost and biocompatible polymer, using a simple protocol enhanced the stability and released of the drug in a controlled manner, which improved drug bioavailability. This polymeric coating enhances the stability of the ZIF-8 structure in acidic medium, which led to targeted drug release in basic media. These finding show that the use ZIF-8 is a good candidate as a targeted delivery system for GLP-1 agonists and will lend a hand to future scientists working in this field to successfully exploit the potential of this drug delivery system for the diabetes therapy. Further study is necessary for the in vitro as well as in vivo drug release study in acidic conditions will be carried out to study to what extent the coated ZIF-8 system will improves the bioavailability of compound **2** and enhance its therapeutic effect (solubility, absorption, bioavailability, and controlled-release of drugs).
